# The Story of the Insane from Year to Year

**Published:** 1894-04-07

**Authors:** 


### THE STORY OF THE INSANE FROM YEAR TO YEAR.

(Sixth Series.)

### WORCESTER ASYLUM.

Here the numbers fell during the year from 918 to 901; but this decrease was not owing to any lack of lunatics, but was caused by the transference of 79 patients who were no longer chargeable to the county. There were 206 admissions, 56 recoveries, and 75 deaths. The recoveries stand at 32·5 per cent. on the admissions, and the deaths at 8·5 on the average number resident. Congenital defect, hereditary influence, and predisposition, taken together, account for 72 of the year's admissions, and drink is credited with the cause in 21. Of the deaths 26 were due to brain disease, and 13 to phthisis. Dr. Cooke draws attention to a fact which has been many a time noted before, namely, the mental improvement which sometimes follows transfer from one asylum to another; and he suggests some alteration in the lunacy law whereby these transfers could be carried out. The idea was brought before the Royal Commission about eighteen years since, but did not find much favour, although no doubt every superintendent has a few patients he would gladly try the experiment on. There are 48 private patients in the asylum, but it does not appear that any special provision is made for them. The committee express their appreciation of the valuable services which the Medical Superintendent continues to render to the institution.

The weekly cost per head is 8s. 4d.

### LEICESTER COUNTY ASYLUM.

Here the numbers went up from 453 to 466. The admissions were 125, the recoveries 41, and the deaths 43. The recoveries work out at 39·80 per cent. of the admissions, and the deaths at 9·49 per cent. on the average number resident. Drink was the cause of the insanity in 13 of the admissions, and hereditary influence in 39. Of the deaths, 17 were caused by brain diseases, and 6 by consumption.

Dr. Higgins is fortunate in being able to record immunity from suicide, serious casualty, or inquest during the year. Many improvements have been carried out, and there would seem to be no lack of amusements for the patients. The weekly rate of maintenance is 9s. 3⅝d.

